# Carbon Nanotube-Mediated Plasmid DNA Delivery in Rice Leaves and Seeds

**DOI:** 10.3390/ijms23084081

**Published:** 2022-04-07

**Authors:** Tia Dunbar, Nikolaos Tsakirpaloglou, Endang M. Septiningsih, Michael J. Thomson

**Affiliations:** Department of Soil and Crop Sciences, Texas A&M University, College Station, TX 77843, USA; tkdunbar@tamu.edu (T.D.); ntsakirp@tamu.edu (N.T.); eseptiningsih@tamu.edu (E.M.S.)

**Keywords:** gene editing, rice (*Oryza sativa*), CRISPR/Cas9, carbon nanotubes (CNTs), *phytoene desaturase* (*PDS*)

## Abstract

CRISPR-Cas gene editing technologies offer the potential to modify crops precisely; however, in vitro plant transformation and regeneration techniques present a bottleneck due to the lengthy and genotype-specific tissue culture process. Ideally, in planta transformation can bypass tissue culture and directly lead to transformed plants, but efficient in planta delivery and transformation remains a challenge. This study investigates transformation methods that have the potential to directly alter germline cells, eliminating the challenge of in vitro plant regeneration. Recent studies have demonstrated that carbon nanotubes (CNTs) loaded with plasmid DNA can diffuse through plant cell walls, facilitating transient expression of foreign genetic elements in plant tissues. To test if this approach is a viable technique for in planta transformation, CNT-mediated plasmid DNA delivery into rice tissues was performed using leaf and excised-embryo infiltration with reporter genes. Quantitative and qualitative data indicate that CNTs facilitate plasmid DNA delivery in rice leaf and embryo tissues, resulting in transient GFP, YFP, and GUS expression. Experiments were also initiated with CRISPR-Cas vectors targeting the *phytoene desaturase* (*PDS*) gene for CNT delivery into mature embryos to create heritable genetic edits. Overall, the results suggest that CNT-based delivery of plasmid DNA appears promising for in planta transformation, and further optimization can enable high-throughput gene editing to accelerate functional genomics and crop improvement activities.

## 1. Introduction

The global population has been projected to reach ten billion by the year 2050; thus, food production must increase by 70% on less total arable land to sustain the human population [1,2]. It remains a fundamental challenge to increase crop yield, resilience, and nutrition if global sustainability is to be achieved. Crops have been improved by selection and traditional breeding, but additional methods are needed, preferably faster, more precise, and less reliant on chance. Advancements in gene editing hold great promise in helping us meet global food demands within a reduced timeframe [3,4]. Genetic transformation methods in plants, however, are far from fully optimized due to cell walls barring delivery of gene-editing components, difficult plant regeneration in vitro, and genotype-specific tissue culture protocols, although recent progress has been made using developmental regulators [5,6,7]. 

This research aims to test and optimize several components of a gene editing system that has the potential to avoid in vitro tissue culture and regeneration and be genotype-independent. Carbon nanotubes (CNTs) have been shown to passively diffuse through plant cell walls and, when loaded with plasmid DNA (pDNA) cargo, can aid in transient expression of foreign DNA in plant cells [8]. CNTs can potentially provide a passive, non-destructive mechanism to transform mature plant embryos with clustered regularly interspaced short palindromic repeats (CRISPR)-associated (Cas) genes to make precise genetic alterations in germline precursor cells. Target gene alterations could be subsequently inherited in progeny, creating an opportunity to select plants with the desired gene edits. In some cases, CNT-mediated gene editing directly into the germline cells through in planta transformation may obviate the need for in vitro tissue regeneration and could potentially be applicable to a wide range of species, offering an ideal platform for high-throughput gene editing applications [9].

The ability of CRISPR-Cas systems to naturally induce targeted double-stranded breaks in host DNA makes it a powerful tool for gene editing. Double-stranded break repair mechanisms are useful for directing not only precise insertion/deletions (indels), but also single-base substitutions, methylation, and acetylation are now possible using fusion proteins with a deactivated Cas9 [3,10,11]. The precision and versatility of the CRISPR-Cas9 system can expand fields such as biological pharmaceuticals [12], gene therapies [13], and agriculture [14,15,16,17], making it a universally essential tool in biotechnology.

Delivery of CRISPR systems still poses a challenge when editing the genome of organisms with cell walls such as plants; methods are labor-intensive and imprecise. For instance, *Agrobacterium tumefaciens*-mediated transformation involves random integration of transgenes, which face tight regulations worldwide [18]. Biolistic approaches, such as particle bombardment, can also be used for plant transformation but are unpredictable and often lead to undesirable effects due to cell damage and random integration of multiple insertions across the genome [19]. Both *Agrobacterium*-mediated and biolistic transformations often involve time-consuming in vitro tissue culturing and regeneration that considerably prolong the gene editing pipeline [20,21]. Transformation protocols involving callus induction are genotype-dependent and pose a cumbersome, lengthy endeavor: plant regeneration from callus takes several months for rice (*Oryza sativa*), even under optimal conditions [22,23]. *Agrobacterium* delivery via floral dip has been shown to allow in planta transformation of some species, although routine use has been limited to the *Brassicaceae* family [24,25]. Circumventing tissue regeneration would facilitate and expedite the transformation process while expanding gene editing to a broader range of cultivars. 

CNTs offer a precise, transient gene editing delivery system that can avoid transgene integration and the subsequent issues dealing with transgene removal. With dimensions as low as 4 nm × 0.5 µm, nanotubes fall below the size exclusion limit of plant cell walls and membranes, allowing them to enter undamaged cells by diffusion through cell walls and penetration through plasma membranes [8]. As visualized by reporter genes, the DNA cargo loaded onto CNTs can be transcribed for several days post-infiltration, proving DNA fragments are able to passively enter cell nuclei when electrostatically grafted to CNTs. Reporter gene expression is transient and diminishes by about ten days post-infiltration [8]. CNTs could potentially serve as a transport mechanism to shuttle CRISPR-Cas machinery into plant cells to create a transient, non-integrative gene editing system. Such a system could directly alter germline cells through in planta transformation, eliminate the challenge of tissue regeneration, and present a genotype-independent method for rapid crop improvement of a wide range of plants. 

The *phytoene desaturase* (*PDS*) gene is a useful target to optimize gene editing: disruption results in albino and dwarf phenotypes [26]. For rice, the Michigan State University (MSU) Rice Genome Annotation database of *Oryza sativa* gene references can be used to identify genes for targeted knockout [27]. *PDS* encodes an oxidoreductase that converts phytoene into zeta-carotene, thus playing a vital role in the biosynthesis of photosynthetic pigments [28]. *OsPDS* has been previously targeted for knockout by gene editing because monoallelic *pds* mutations are generally nonlethal, have been replicated by *Agrobacterium*-mediated transformation, and produce an easily recognizable dwarfed-albino phenotype that acts as a visual indicator of successful gene editing [29,30]. Single guide RNAs (sgRNAs) complementary to MSU’s 5.7 kb *OsPDS* (LOC_Os03g08570.1) sequence from *O. sativa subsp. japonica* can be designed and verified in vitro for subsequent gene knockout experiments. The *OsPDS* knockout genotypes can be aligned to the wild type reference genome sequence to assess indels [27].

CNT capacity to passively transport pDNA cargo in planta has been documented by Demirer et al. [8], but not in rice. The current study provides evidence verifying the ability of CNTs to transport reporter plasmids into mature rice leaves and seeds. Successful transport has been identified by visual cues and molecular data specific to each reporter gene: green fluorescent protein (GFP), yellow fluorescent protein (YFP), and β-glucurodinase (GUS). Further, transient transformation of rice seeds by passive diffusion of CNT-plasmid complexes containing the CRISPR-Cas9 elements targeting the *OsPDS* resulted in detection of mutations in the expected target areas. This study enables the establishment of a testing platform for further optimization of CNT-based protocols towards high-throughput gene editing.

## 2. Results

### 2.1. Transient Fluorescence in Rice Leaves and Embryos after CNT-Mediated Plasmid Delivery

**Figure 1:**
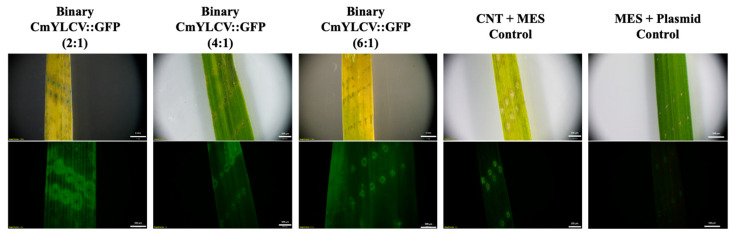
Brightfield and fluorescence stereomicroscope images of rice leaves three days post-infiltration with binary CmYLCV::GFP pDNA-CNT solutions at 2:1, 4:1, or 6:1 pDNA:CNT ratios. Exposure time was 800 ms.

**Figure 2:**
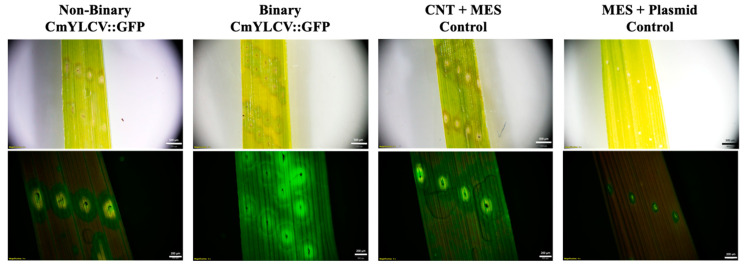
Brightfield and fluorescence stereomicroscope images of rice leaves three days post-infiltration using binary and nonbinary vectors encoding GFP with CmYLCV promoters. The CNT solution consists of a 2:1 pDNA:CNT ratio. Exposure time was 800 ms.

CNTs were functionalized with polyethylenimine (PEI) and attached with pDNA as directed by Demirer et al. [31]. Leaves were first punctured with needles and submerged in MES delivery buffer with GFP-expressing pDNA-CNTs at concentrations of 1.5 ng pDNA per µL and at ratios ranging from 6:1 to 2:1 pDNA:PEI-CNTs. Images were taken with a stereomicroscope after three days in solution. A comparison across the 2:1, 4:1, and 6:1 ratios of plasmid DNA to CNTs showed that the 2:1 ratio had the highest level of fluorescence (Figure 1). A 2:1 ratio was therefore used in subsequent infiltrations investigating the effects of plasmid cargo size. Increased fluorescence can be seen in leaves treated with binary (10.5 kb) or nonbinary (3.7 kb) GFP plasmid vectors when compared to CNT-only and plasmid-only controls (Figure 2). These results suggest that plasmid DNA up to 10.5 kb in size was successfully delivered and expressed in rice leaf cells by the CNTs, and then transcribed and translated in the cells, leading to the fluorescent signal from the GFP reporter gene.

**Figure 3:**
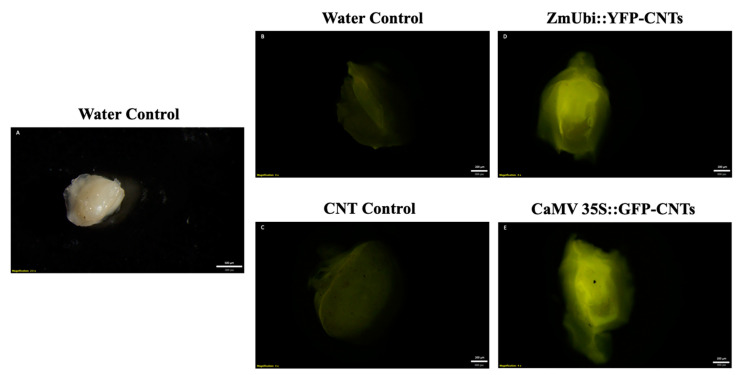
Brightfield and fluorescence stereomicroscope images of excised rice embryos two days post-imbibement: (**A**) water control (brightfield); (**B**) water control (fluorescence); (**C**) CNT-only control; (**D**) ZmUbi::YFP-CNTs; and (**E**) CaMV 35S::GFP-CNTs. Solutions consist of 1:3 pDNA:CNT ratios. Fluorescence was observed through a GFP filter at an exposure time of 125 ms.

Further, excised embryos were imbibed in pDNA-CNT solutions for two days prior to viewing fluorescence under the microscope. Although some background fluorescence was present in the control samples, an increase in fluorescence can be seen in the samples imbibed with CNTs attached with CaMV::GFP and ZmUBI::YFP plasmids at a 1:3 ratio of plasmid DNA to CNTs (Figure 3). 

**Figure 4:**
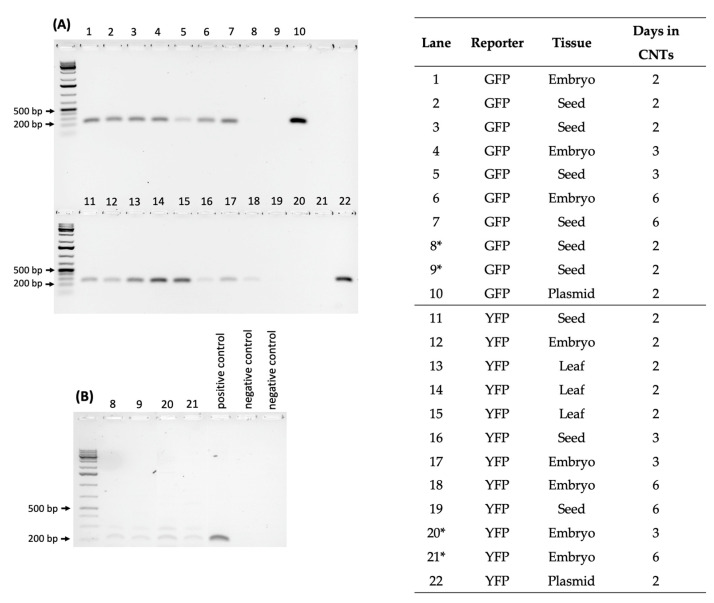
RNA was extracted from GFP- or YFP-CNT-treated leaf, embryo, and seed tissues, and complementary DNA (cDNA) was synthesized. (**A**) cDNA was amplified with GFP- or YFP-specific primers following KAPA3G Plant PCR Kit parameters. Expected fragment sizes were 215 bp and 238 bp for GFP and YFP primer sets, respectively. Gel lane numbers with the corresponding reporter gene, source tissue, and number of days submerged in CNT solution are shown at the right; samples with an asterisk did not amplify a detectable product. (**B**) As an additional precaution to verify proper RNA extraction and cDNA synthesis from the samples with negative results, primers of the housekeeping gene OsActin1 were used to PCR amplify cDNA from samples from lanes 8, 9, 20, and 21 in (**A**) above, showing successful amplification of OsActin1 from these four samples, although at a low level (expected fragment size: 195 bp). Ladders: GeneRuler 1 kb Plus (Thermo Fisher Scientific, Catalog No. SM1331).

Moreover, reverse transcription PCR (RT-PCR) with GFP-specific primers on a subset of fluorescing samples showed three out of three excised embryo samples showing a positive product, while four out of six whole seeds tested positive for GFP transcripts. Similarly, three out of five excised embryos and three out of three imbibed seeds tested positive for YFP-specific primers (Figure 4). Although further optimization is needed, these results showed promise for the ability of CNTs to deliver plasmids into excised embryos.

**Figure 5:**
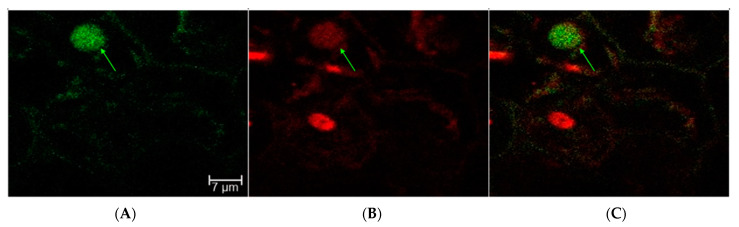
Excised leaves and embryos treated with CNTs carrying NLS-GFP vectors were sectioned, stained with DRAQ5, and imaged under confocal microscope after four days in solution. (**A**) GFP filter, (**B**) DRAQ5 filter, and (**C**) a and b overlay. Image overlays indicate GFP fluorescence overlap with nuclear staining. Sectioning and imaging performed by TAMU MIC.

To test whether a nuclear-localized GFP may provide more clear fluorescence signal over the background fluorescence, leaves and excised embryos were infiltrated with a nuclear-localized GFP (NLS-GFP) grafted to CNTs. The sectioning, staining, and imaging of NLS-GFP-CNT-treated leaves and embryos were performed at the Texas A&M University (TAMU) Microscopy and Imaging Center (MIC) (https://microscopy.tamu.edu/, accessed on 5 April 2021). Due to unforeseen setbacks, however, samples were left at unfavorable conditions for an additional 24 h and were not imaged until four days post-CNT treatment. Despite fluorescent noise, results showed some nuclei with fluorescence, indicating successful plasmid NLS-GFP delivery and expression (Figure 5).

### 2.2. Transient GUS Expression after CNT-Mediated Plasmid Delivery

**Figure 6:**
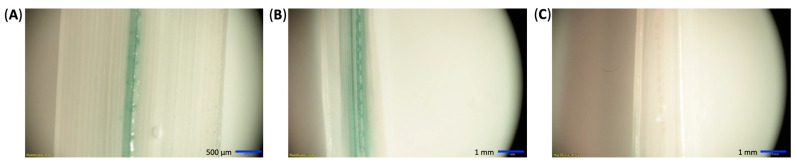
Rice leaves were punctured with needles and imbibed in CNTs loaded with (**A**) nonbinary or (**B**) binary GUSPlus vectors. After three days in solution, GUSPlus enzymatic activity was visualized by histochemical assay [32] and chlorophyll bleaching. Blue coloration indicates GUSPlus activity at the infiltration site of treated leaves. (**C**) Lack of blue color in a negative control leaf for comparison.

**Figure 7:**
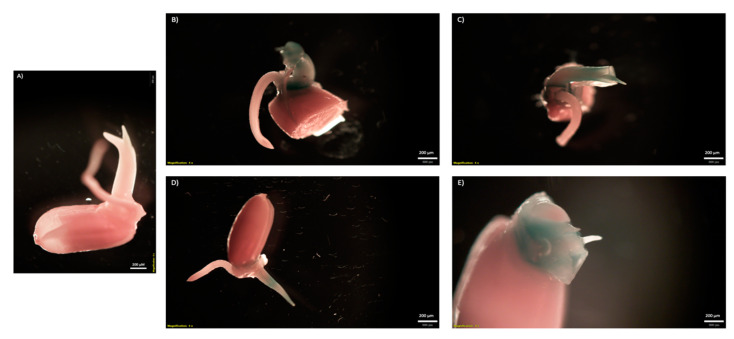
Histochemical detection of GUS expression in rice seeds imbibed in a CNT-plasmid DNA solution. Sterilized rice seeds were soaked overnight in water to initiate germination, then with a osmotic solution of 0.6 mannitol for two hours, followed by a 5-day exposure to a CNT-pDNA solution at 1:3 ratio with a 35S::GUSPlus vector. GUSPlus enzymatic activity was visualized by histochemical assay using standard procedures [32]. (**A**) Imbibed rice seed in MES delivery buffer (negative control). (**B**–**E**) Detection of GUS expression in germinating rice tissues after 5 days in the CNT-pDNA mixture solution.

To further confirm the leaf infiltration results seen with the GFP plasmids, a GUSPlus assay was also performed. Binary and nonbinary vectors encoding GUSPlus enzymes were also used to visualize transient expression facilitated by CNTs. Leaves were punctured and imbibed for three days with the pDNA-CNT solution to allow a maximum accumulation of GUSPlus transcripts. Leaves were fixed in formaldehyde, subjected to histochemical assay, and leached of chlorophyll. Successful pDNA-CNT delivery into rice leaf tissue can be seen in the blue coloration in the treated rice leaves (Figure 6). The images from Figure 6 were taken after 24 h once all chlorophyll had been removed. Blue coloration indicative of GUSPlus enzymatic activity was only visible in GUSPlus-treated samples, while negative controls remained colorless. Although the GUSPlus activity was most visible at the puncture sites where CNTs were able to infiltrate, it also seemed to follow the vein pattern along the leaves. Likewise, a GUSPlus assay was performed to test CNT-mediated delivery of plasmid DNA in germinating rice seeds. Sterilized rice seeds were soaked overnight in water to initiate germination, and then in a 0.6 M mannitol osmotic solution for two hours prior to the exposure to the CNT-pDNA mixture for 5 days. Histochemical detection of GUS expression was performed following standard procedures. The binary vector CGEL-001 (pCambia 1305.1) with 35S::GUSPlus was used for the plasmid DNA. Clear evidence of GUS expression in tissues growing from the embryo was observed (Figure 7). 

### 2.3. Testing CNT Delivery of CRISPR-Cas for Gene Editing of Rice Seeds and Embryos 

To test whether CRISPR-Cas reagents can be delivered into plant tissues by CNTs and transiently expressed to provide gene editing in germline cells, two approaches were tested: imbibing whole seeds in a CRISPR-Cas-CNT solution or excised mature embryos (to expose the shoot apical meristem (SAM)) and imbibing in the CNT solution. OsPDS was selected as a target gene for testing, because OsPDS knockouts are known to produce stunted, albino seedlings. CRISPR-Cas-CNT-imbibed seeds and embryos were grown on plant nutrient medium (MS0) for up to two weeks to view alterations in the phenotype indicative of OsPDS knockout. A total of 1120 seeds and 112 embryos were imbibed in CRISPR-Cas-CNT solution, and a total of 121 (10.8%) seeds and 13 (11.6%) embryos displayed phenotypic abnormalities. Seedlings with partially albino leaves and/or stunting were selected for further study (Supplementary Figure S1, see Supplementary Materials). 

### 2.4. DNA Sequencing of Phenotypically Aberrant Seedlings 

Sanger sequencing of PCR products targeting exon 3 of *OsPDS* was performed to identify potential edits at the target site. Due to the difficulty in analyzing Sanger sequencing data potentially consisting of mixed reads with insertions/deletions of varying lengths, the Synthego Inference of CRISPR Edits (ICE) software tool was used to analyze the Sanger sequencing chromatograms of *OsPDS*. Out of the total of 1120 seeds that were imbibed in CRISPR-pDNA-CNT solutions targeting *OsPDS*, seedlings displaying even minute phenotypic differences, such as stunted growth or discolored leaves, were used for subsequent *OsPDS* sequencing analysis. DNA was extracted from a total of 121 (10.8%) phenotypically aberrant seedlings. Synthego’s ICE tool was used to batch analyze Sanger trace files [33]. Out of all sequencing data covering the sgRNA1 and sgRNA2 regions, 33 showed putative indels in the sgRNA1 target region, while 9 showed putative indels in the sgRNA2 region when specifying a minimum R^2^ threshold of 0.7 with greater than 5% indel percentage. Although the ICE analysis appeared to identify putative insertion/deletion mutations at the target site, overall, the sequencing results were inconclusive. For example, one of the representative ICE alignments for the two *OsPDS* sgRNAs showed the normalized relative contribution of a single base pair deletion with a 9% contribution, the wild type at 52%, and a 12 bp insertion contributing 3%, which was below the threshold (Supplementary Figure S2). A set of 105 samples were also sequenced using target amplicon sequencing with an Illumina MiSeq, resulting in 6 samples showing a 1 bp deletion at sgRNA1, 20 samples showing a 1 bp deletion at sgRNA2, and 1 sample with a 1 bp insertion at sgRNA2, albeit in the heterozygous or chimeric state and at low frequencies versus the wild type alleles (Supplementary Figure S3; Supplementary Table S1). In addition to the small insertion and deletions, a number of base substitutions were observed, but only the insertion/deletions led to frameshift mutations and premature stop codons (Supplementary Figure S4).

## 3. Discussion

Recent studies have demonstrated that carbon nanotubes (CNTs) loaded with plasmid DNA can diffuse through plant cell walls, but this has not yet been confirmed in rice [6,26]. The current study aimed to test several parameters for CNT-mediated plasmid DNA delivery into rice tissues, starting with reporter genes and leaf infiltration, with the end goal of using CNT delivery of gene editing reagents into seeds for rapid in planta transformation. The experiments collectively evaluated a variety of fluorescent-reporter plasmids that differed in size, gene promoter, and concentration relative to CNTs. Three pDNA:CNT ratios (2:1, 4:1, and 6:1) were investigated for the relative efficiency of CNT-mediated delivery using rice leaf infiltration. Fluorescence was not obvious in leaves treated with the higher ratios of plasmid to CNTs, and the lowest ratio, 2:1, produced the most fluorescence. While the 2:1 ratio lies nearly in the middle of the range presented by Demirer et al. [31], the optimal ratio may differ across plant species.

To test if plasmid size affected the efficiency of CNT delivery into plant cells, both large plasmids (binary vectors originally developed for *Agrobacterium*-mediated transformation) and small (non-binary) plasmids were used, modified from a published gene editing toolkit [34]. Fluorescence imaging revealed a higher signal from both binary and nonbinary CmYLCV::GFP-CNT-treated leaves than the negative control leaves. Although it would be expected that the small size of the non-binary vectors would facilitate passive movement into plant cells, the non-binary vectors did not produce visibly noticeable differences in fluorescence compared to binary plasmids driven by the same promoter.

After confirmation of CNT-mediated delivery into leaves, the applicability of CNT passive diffusion into rice embryos was examined using YFP and GFP reporter genes. Assessments by imaging and transcript analysis strongly suggest transcription and translation of the reporter plasmids in imbibed leaves, seeds, and embryos. Additionally, amplification of housekeeping genes in negative control complementary DNA (cDNA) samples confirms successful RT-PCR across samples. Moreover, successful expression of a nuclear-localized GFP also supported the efficacy of CNT-mediated delivery. Likewise, a GUS assay also demonstrated reporter gene expression after CNT-mediated delivery of plasmid DNA in rice leaves and in imbibed seeds.

Promising findings with reporter genes provided support to begin testing nanoparticles for initial in planta transformation experiments for gene editing. Plasmids containing Cas9 and two guide RNAs targeting *OsPDS* were delivered into imbibed whole seeds using CNT-mediated delivery, and stunted, yellowing phenotypes were noted in a subset of treated seedlings. The subsequent sequence analysis indicated potentially chimeric tissues and a very low frequency of mutations. Follow up studies can further optimize this protocol in several areas; vacuum infiltration parameters such as time and pressure can be investigated for effects on transformation efficiency. Different promoters driving sgRNA expression can also be explored. The amount of sgRNA:Cas9 complexes within the cells can also be enhanced, for instance by using a combination of the PDS cassette with self-replicating plasmids and CNT delivery. Future experiments should also investigate editing efficiency in germline cells and precursors by assessing the heritability of edits in progeny, and test larger numbers of seeds to detect low frequency editing events. 

Overall, the preliminary results from the reporter gene experiments seem to indicate that loaded CNTs are capable of entering leaf tissue and traversing the seed coat. Although this protocol needs further optimization, future efforts can potentially improve this nanotechnology-based CRISPR-Cas9 delivery approach into a straightforward, non-transgenic, in planta gene editing system that can bypass the tissue regeneration bottleneck of conventional methods.

## 4. Materials and Methods

### 4.1. Plant Materials

All the rice materials that were used in this study were from cv. Nipponbare or cv. Presidio and were grown in standard MS medium in growth chambers (14-h light, 10-h dark cycles at 29 °C and 25% relative humidity), unless stated otherwise.

### 4.2. Plasmid-PEI-CNT Preparation

PEI-CNTs were prepared as directed by the protocol from Demirer et al. [31] to functionalize the CNTs in preparation for attachment of the plasmid DNA. Briefly, single-walled, carboxylic acid functionalized carbon nanotubes (Cat. no. 652490, Sigma-Aldrich, St. Louis, MO, USA) were first covalently modified with the cationic polymer polyethylenimine (PEI, branched, molecular weight 25,000; Cat. no. 408727, Sigma-Aldrich, St. Louis, MO, USA) to carry a net positive charge in preparation for attaching plasmid DNA. Following the published protocol, zeta potential was measured by Zetasizer Nano ZS (Malvern Panalytical, Malvern, UK) and determined within the appropriate +50 to +70 mV range before continuing. Fresh PEI-CNTs were stored in aliquots at 5 °C and prepared fresh every month or until agglomeration of nanoparticles became visible to the naked eye. 

Fresh pDNA was electrostatically grafted to PEI-CNTs following the protocol of Demirer et al. [31]. Chilled CNT aliquots were brought to room temperature 30 min prior to a 45-min bath sonication to thoroughly resuspend nanoparticles. CNTs were diluted to appropriate concentrations in 2-(N-morpholino)-ethanesulfonic acid (MES) delivery buffer (Sigma-Aldrich, St. Louis, MO, USA). pDNA-PEI-CNT solutions were prepared in varying pDNA-to-CNT ratios ranging from 6:1 to 1:3. Activated PEI-CNTs were added to pDNA at least 30 min prior to use in experiments.

### 4.3. Plasmid Preparation

Reporter plasmids ranged in size from nonbinary vectors fewer than 3.7 kb to binary gene editing vectors of nearly 12.0 kb. Six main reporter plasmids were tested to visualize CNT transformation (Table 1). Binary and non-binary versions of the prCmYLCV::GFP cassette were prepared following the procedure previously described [34]. The intermediate modular vectors that were included for the Golden Gate assembly were the CGEL-011 (or pMOD_A3003, Addgene: #91043; Watertown, MA, USA), the CGEL-012 (or pMOD_B0000; Addgene: #91058), and the CGEL-020 (or pMOD_C0000, Addgene: #91081); backbone vectors were either the CGEL-004 (or pTRANS_100; Addgene: #91198) or the CGEL-005 (or pTRANS_210, Addgene: #91108). To prepare the nonbinary version of the pr35S::GUSPlus cassette, the NT057 and NT058 primer sequences were used to amplify the pr35S::GUSPlus:NOS 3′UTR (pCAMBIA1305.1, GeneBank: AF354045.1) using Phusion High-Fidelity DNA polymerase (Cat #F530L, ThermoFisher Scientific, Waltham, MA, USA), whilst introducing the HindIII and BamHI restriction sites at the 5′ and 3′ ends of the cassette. These restriction sites were used for directional cloning of the fragment into pUC19 (GeneBank: M77789.2), and the correct orientation of the final plasmid was confirmed by sequencing. Similarly, for the preparation of the nonbinary version of the vector containing the sgRNAs targeting the *PDS* gene in rice, the following strategy was followed. Initially the tRNA-sgRNA fragment containing the sgRNAs targeting the *OsPDS* gene was amplified from the previously prepared binary version of the vector [35] using the NT062 and NT063 primers, whilst introducing the HindIII and BamHI restriction sites in either site of the fragment. Moreover, Esp3I restriction sites generating overhangs compatible to the CGEL-018 (or pMOD_B2519, Addgene: #91076) also flanked the tRNA-sgRNA fragment. The fragment was initially TOPO cloned in pCR Blunt II TOPO vector, and the sequence was confirmed by sequencing. Subsequently, the fragment was subcloned to CGEL-018 through directional cloning utilizing the compatible overhangs generated by Esp3I restriction digestion. Finally, the nonbinary version of the plasmid was assembled by Golden Gate cloning, as described previously. Other components of the Golden Gate reaction included the CGEL-004 plasmid, the CGEL-008 plasmid (or pMOD_A1110, Addgene: #91031), and the CGEL-020 plasmid. 

CRISPR gene editing plasmids were nonbinary plasmids only 9.3 kb in size (Table 1). The gene editing cassette contained two sgRNAs (sgRNA1 and sgRNA2) that were previously designed to target Exon 4 and Exon 3 of *OsPDS*, respectively [35]. Each sgRNA was previously verified by in vitro assay using cv. Nipponbare and Presidio genomic DNA.

### 4.4. Leaf Infiltrations with CNTs

Maturing rice plants were grown in a Conviron chamber (Pembina, ND, USA) under long-day conditions (14-h light, 10-h dark cycles at 29 °C and 25% relative humidity) until at least three true leaves had emerged. Leaves were mechanically wounded with needles and soaked in pDNA-PEI-CNT solution as described for monocot leaf infiltration [36]. Binary and nonbinary pDNA vectors encoding YFP, GFP, or GUSPlus driven by different constitutive plant promoters, as depicted in Table 1, were infiltrated into the leaves. Plants were kept at room temperature and ambient lighting during infiltration to preclude the possible breakdown of fluorescent proteins under extreme light conditions [37]. Fluorescence was observed by an Olympus SZX10 stereomicroscope (Waltham, MA, USA), and GUS enzymatic activity was visualized by histochemical assay [32] following 24-h intervals post-infiltration.

### 4.5. Rice Embryo Infiltrations with CNTs and Reporter Plasmids

Prior to treatment with CNTs, mature desiccated rice seeds were surface sterilized in a 70% ethanol solution for three minutes, followed by a 4.5% bleach wash for 35 min [38,39]. Seeds were thoroughly washed five times with autoclaved deionized water to remove bleach. To initiate germination, surface-sterilized seeds were placed in a petri dish, covered with sterilized water, and incubated overnight at 29 °C.

Mature embryos were excised from sterilized seeds. Shoot tips were cut away to reveal SAMs, and the embryos were cut away from the endosperm using a fine-point scalpel. Such methods have been previously employed to facilitate the production of transgenic plants [40,41]. Excised embryos were placed in a 0.6 M mannitol osmotic solution for two hours prior to drying on sterile filter paper and submergence in pDNA:CNT solutions at varying ratios.

Plasmids were covalently attached to positively charged CNTs at least 30 min prior to use. Embryos were vacuum-infiltrated at 500 mm Hg for five minutes in CNT solution before storing at 29 °C on a 14-h light, 10-h dark cycle. Images were taken at 24-h intervals by Olympus SZX10 stereomicroscope (Waltham, MA, USA).

### 4.6. Transcript Analysis

GFP and YFP expression was confirmed via RT-PCR analysis. Fluorescing leaves were excised and immediately frozen by liquid nitrogen. A SPEX 1600 MiniG plate homogenizer (Metuchen, NJ, USA) was used to grind leaf tissues into a fine powder before extracting RNA following instructions from a Qiagen RNeasy Plant Mini Kit (Germantown, MD, USA). RNA was treated with DNase and cDNA was synthesized with an Invitrogen SuperScript ™ III First-Strand Synthesis System and oligo (dT) primers (ThermoFisher Scientific, Waltham, MA, USA). The resulting cDNA was treated with RNase prior to PCR amplification (KAPA3G Plant PCR Kit, (MilliporeSigma, Burlington, MA, USA)) with transcript-specific primers (Supplementary Table S2). Primers specific to the rice housekeeping gene OsActin1 (LOC_ Os05g36290) were used to PCR amplify cDNA of negative controls to verify proper cDNA synthesis [42]. PCR products were separated by agarose electrophoresis (1.5% (g/v), 75 min at 50 V) to visually confirm the presence or absence of amplicons indicative of GFP or YFP transcripts in leaf extracts.

### 4.7. Confocal Imaging of NLS-GFP

Binary plasmids encoding a plant-specific nuclear-localized GFP were also tested with CNT infiltration of rice leaves and embryos. Tissue samples were brought to the TAMU MIC for hand-sectioning, staining, and imaging of both leaf and embryo samples. Nuclei were stained with DRAQ5 to delineate plant nuclei according to Smith et al. [43], and images were taken under a Leica Microsystems SP8 confocal microscope (Buffalo Grove, IL, USA).

### 4.8. GUSPlus Analysis

Imbibed leaves were fixed in formaldehyde, underwent Cervera’s GUS histochemical assay protocol [32], and de-stained with ethanol. GUSPlus expression in transformed tissues was confirmed via transcript analysis: leaves treated with GUSPlus vectors and CNTs were ground into a fine powder and processed by Qiagen RNeasy Plant Mini Kit (Germantown, MD, USA) and Invitrogen SuperScript™ III First-Strand Synthesis System (ThermoFisher Scientific, Waltham, MA, USA). cDNA was PCR amplified with GUSPlus-specific primers. Primers specific to the housekeeping gene LOC_Os03g08020 encoding RICE ELONGATION FACTOR1-ALPHA-LIKE (EF1a) were used to amplify cDNA as a positive reverse-transcription control. cDNA was separately amplified by PCR with primers specific to the selective marker of the plasmid backbone as a control, ensuring proper DNase treatment. All in-house primer sequences can be found in Supplementary Table S2. PCR products were run on a 1.5% agarose gel to visually confirm the presence or absence of amplicons indicative of GUSPlus transcripts in leaf tissues. Likewise, histochemical detection of GUS expression was performed in rice seeds imbibed in CNT-plasmid DNA solution at a 1:3 ratio. Sterilized rice seeds were soaked overnight in water to initiate germination and then in osmotic solution prior to the exposure to CNT-pDNA mixture for 5 days. Histochemical detection of GUS expression was performed following standard procedures. The binary vector CGEL-001 (pCambia 1305.1) was used as plasmid DNA.

### 4.9. CRISPR/Cas9-CNT Seed and Embryo Infiltration

Mature Nipponbare and Presidio seeds were surface-sterilized and germinated as described in previous sections. Seeds were mechanically wounded to expose the shoot apical meristem (SAM) and subsequently imbibed in a 0.6 M mannitol osmotic solution for at least two hours. Seed tissues were briefly dried on sterile filter paper, then imbibed in a CRISPR-Cas9 pDNA-CNT solution as described previously. Imbibing solutions were concentrated to 1.5 ng/µL of plasmid DNA encoding Cas9 and sgRNAs targeting *OsPDS*. Samples were vacuum-infiltrated at 500 mm Hg for up to five minutes and stored at 29 °C on a 14-h light, 10-h dark cycle. Selected seeds were removed from CNT solutions at 24-h intervals and plated on MS0 agar [44] to form roots and shoots. Plants were grown for at least two weeks to observe possible *OsPDS* knockout phenotypes, which appear as albino and stunted seedlings.

### 4.10. OsPDS Knockout Sequencing Analysis

A quick preliminary screen for *OsPDS* mutations was performed by Sanger sequencing. Genomic DNA samples were extracted from pDNA-PEI-CNT-treated seedlings using a small-scale CTAB method [45]. Genomic DNA was PCR-amplified by Phusion High-Fidelity DNA polymerase (Thermo Fisher Scientific, Waltham, MA, USA) using primers flanking the *OsPDS* region targeted by the sgRNAs (Supplementary Table S2). PCR amplicons were separated by agarose electrophoresis, extracted by Zymoclean Gel DNA Recovery Kit (Zymo Research, Irvine, CA, USA), and submitted for Sanger sequencing to either the TAMU Laboratory for Genomic Technology (LGT) or Eurofins (https://eurofinsgenomics.com/en, accessed on 30 April 2021). 

The resulting .ab1 sequence trace files were initially batch-analyzed by Synthego’s ICE tool at each sgRNA site [46]. ICE was able to distinguish multiple sequenced fragments within trace files, allowing quantification and characterization of edits within samples. Because ICE cannot discern indels from noise when present at less than 5%, samples that fell below this 5% threshold were discounted [47]. A minimum R^2^ threshold was chosen at 0.7 to minimize error while still identifying low-frequency mutations.

Likewise, samples for target sequence analysis using next-generation sequencing were prepared using genomic DNA or previously isolated PCR product as the template. The respective fragments were amplified incorporating the Illumina Nextera DNA UD Indexes (underlined) to the target region specific primers (OsPDS_sgRNA1_NGS_F (5′- TCGTCGGCAGCGTCAGATGTGTATAAGAGACAGTTCACTGTTAGTAGCATTTGTGG -3′) primer and OsPDS_sgRNA1_NGS_R primer (5′- GTCTCGTGGGCTCGGAGATGTGTATAAGAGACAGCACAACGAGAACCTTAAAAATCCAG -3′) and OsPDS_sgRNA2_NGS_F (5′- TCGTCGGCAGCGTCAGATGTGTATAAGAGACAGTCGTGATTGCTGGAGCAGGTA -3′) primer and OsPDS_sgRNA2_NGS_R primer (5′- GTCTCGTGGGCTCGGAGATGTGTATAAGAGACAGCCTTTCCACCCAAAACATCCCT -3′)). Illumina adapter sequences (i7 and i5) were added subsequently. Pair-end target amplicon analysis (1 million read pairs (Read Length-250 × 250)) was performed through MiSeq, and the resulted fastq files were analyzed by CRIS.py.

## 5. Conclusions

Genome editing will be an important tool for adapting crops to future production challenges. Current methods, however, are constrained by bottlenecks such as tissue culturing and plant regeneration. The current study aimed to make the first steps towards bypassing the tissue culture process altogether by taking advantage of nanoparticles, specifically carbon nanotubes (CNTs). CNTs are non-toxic to biological systems at low concentrations, are capable of electrostatically attaching to DNA, and fall below cell wall and cell membrane size exclusion limits. CNTs may prove valuable as their passive diffusion across cell walls and membranes offers a genotype-independent mechanism to transport CRISPR vectors for in planta gene editing. 

The current study found that CNTs are capable of traversing plant cell walls to deliver reporter plasmids in intact rice tissues. Transient expression of reporter genes acted as visual indication of effective CNT diffusion through intact cell walls as well as successful transcription and translation of foreign genetic elements from within the plant cells. Evidence was obtained from two reporter systems—fluorescent proteins and GUSPlus proteins—both expressed in planta after pDNA-CNT imbibement. CNTs were able to transport both binary and non-binary plasmids, and transcription was validated by cDNA analysis to further confirm visual indicators. Though further experimentation and protocol modifications will be needed to further optimize these protocols, these preliminary results suggest that CNTs show promise as a tool for in planta gene editing in rice.

## Figures and Tables

**Table 1:** List of plasmid information.

Vector Backbone	Reporter Gene	Promoter	Size (kb)
pPTN (binary)	YFP	ZmUbi	12.0
pCAMBIA1305.1 (binary)	GUSPlus	CaMV 35S	11.8
pTRANS210 (binary)	GFP	CmYLCV	10.5
pTRANS100 (nonbinary)	OsPDS sgRNA, Cas9	OsU3, ZmUbi	9.3
pUC19 (nonbinary)	GUSPlus	CaMV 35S	5.4
pENTR (nonbinary)	Nuclear-Localized GFP	EL2	5.1
pTRANS100 (nonbinary)	GFP	CmYLCV	3.7

## Data Availability

The data presented in this study are available within the published article.

## Supplementary Materials

The following supporting information can be downloaded at: https://www.mdpi.com/article/10.3390/ijms23084081/s1.
