# Supplementary material for: Carbon Nanotube-Mediated Plasmid DNA Delivery in Rice Leaves and Seeds
Source: Int J Mol Sci. 2022 Apr 7;23(8):4081. doi: 10.3390/ijms23084081 (PMC9028948; doi:10.3390/ijms23084081)
Supplement: Supplementary file 1 [file ijms-23-04081-s001.zip › ijms-1599492-supplementary.pdf]

Supplementary Materials

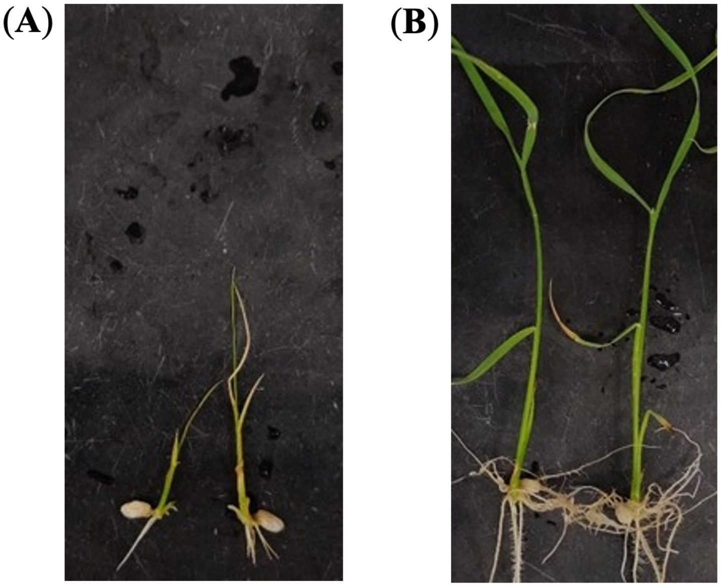

Supplementary Figure S1. Subset of rice seeds that were imbibed in pDNA-CNT solution at a 2:1 CRISPR/Cas vectors to CNT ratio. Seeds were grown on sterile MS0 media plates for up to two weeks. Experimental seeds (**A**) that showed altered growth compared to (**B**) healthy, wild type seeds, were used for subsequent sequencing experiments.

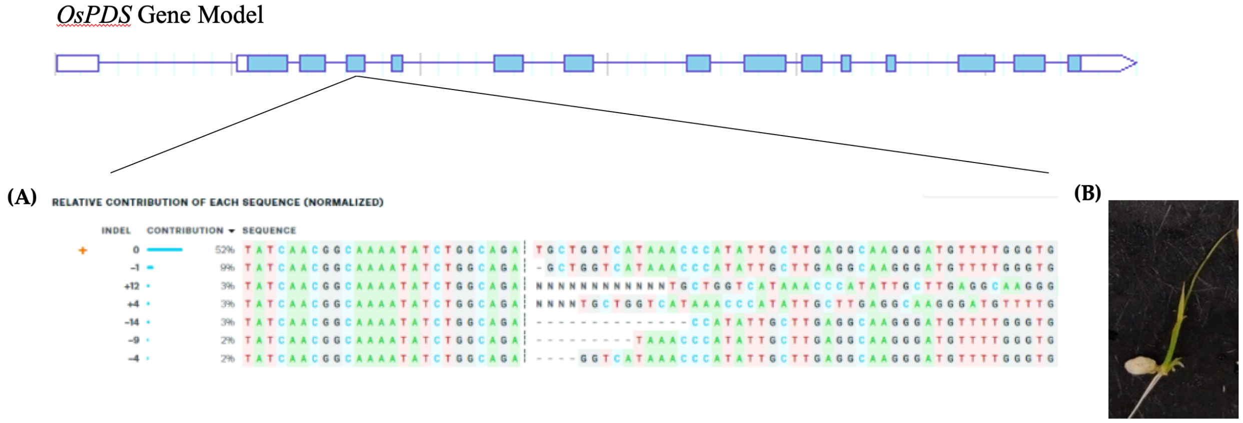

Supplementary Figure S2. Synthego ICE analysis tool was run on individual trace files returned from Sanger sequencing of potential mutant rice seedlings. (**A**) Sequences present in the edited population and their relative proportions. Expected cut sites three bp upstream the PAM are represented by black vertical dotted lines, and the wild type sequence is marked by the orange "+" symbol. (**B**) Example of a phenotypically aberrant seed sent for Sanger sequencing. OsPDS gene model from MSU Rice Genome Annotation database.

sgRNA2, 1 bp insertion
R1 – 1,205 reads, frequency: 0.08% (1/1205)

```
AGGATTAGCTGGTTTATCAACGGCAAAATATCTGGCAGATGCTGGTCATAAACCCATATTGCTTGAGGCAAGG , 1162
AGGATTAGCTGGTTTATCAACGGCAAAAATATCTGGCAGATGCTGGTCATAAACCCATATTGCTTGAGGCAAGG , 1
```

R2 – 1,218 reads, frequency: 0.08% (1/1218)

```
CAATATGGGTTTATGACCAGCATCTGCCAGATATTTTGCCGTTGATAAACCAGCTAATCCTGAAAAAAAAATC , 1187
CAATATGGGTTTATGACCAGCATCTGCCAGATATTTTTGCCGTTGATAAACCAGCTAATCCTGAAAAAAAAATC , 1
```

sgRNA2, 1 bp deletion
R1 – 1,317 reads, frequency: 0.2% (3/1317)

```
AGGATTAGCTGGTTTATCAACGGCAAAATATCTGGCAGATGCTGGTCATAAACCCATATTGCTTGAGGCAAGG , 1249
AGGATTAGCTGGTTTATCAACGGCAAAATCTCTGGCAGATGCTGGTCATAAACCCATATTGCTTGAGGCAAGG , 3
AGGATTAGCTGGTTTATCAACGGCAAA-TATCTGGCAGATGCTGGTCATAAACCCATATTGCTTGAGGCAAGG , 3
```

R2 – 1,258 reads, frequency: 0.2% (3/1258)

```
CAATATGGGTTTATGACCAGCATCTGCCAGATATTTTGCCGTTGATAAACCAGCTAATCCTGAAAAAAAAATC , 1250
CAATATGGGTTTATGACCCGCATCTGCCAGATATTTTGCCGTTGATAAACCAGCTAATCCTGAAAAAAAAATC , 3
CAATATGGGTTTATGACCAGCATCTGCCAGATA-TTTGCCGTTGATAAACCAGCTAATCCTGAAAAAAAAATC , 3
```

sgRNA2, 1 bp deletion

R2 – 1,409 reads, frequency: 0.1% (3/2513)

```
CAATATGGGTTTATGACCAGCATCTGCCAGATATTTTGCCGTTGATAAACCAGCTAATCCTGAAAAAAAAATC , 2482
CAATATGGGTTTATGACCAGCATCTACCAGATATTTTGCCGTTGATAAACCAGCTAATCCTGAAAAAAAAATC , 4
CAATATGGGTTTATGACCAGCATCTGCCAGATATTTTACCGTTGATAAACCAGCTAATCCTGAAAAAAAAATC , 4
CAATATGGGTTTATGACCAGCATCCGCCAGATATTTTGCCGTTGATAAACCAGCTAATCCTGAAAAAAAAATC , 3
CAATATGGGTTTATGACCAGCATCTGCCAGATA-TTTGCCGTTGATAAACCAGCTAATCCTGAAAAAAAAATC , 3
```

Supplementary Figure S3. Sequence analysis of the target amplicon results for the sgRNA-2 region, with the PAM underlined and the PAM plus wild type gRNA sequence highlighted in yellow. All the detected mutations were either heterozygous or chimeric. Insertions and deletions were detected at frequencies ranging from 0.08% to 0.2% (MiSeq read counts are shown in right column). Several base substitutions in the target sgRNA-2 region are also shown.

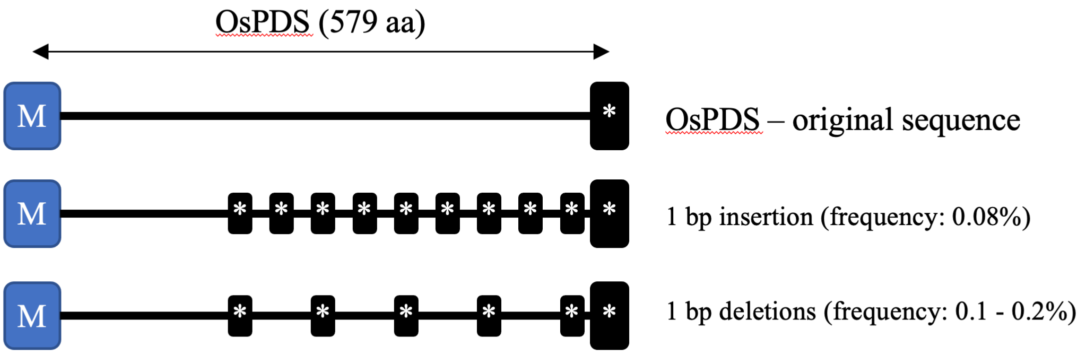

Supplementary Figure S4. Graphical representation of the stop codon peptides created in the OsPDS protein as a consequence of the 1 bp insertion or 1 bp deletions in the target region of sgRNA-2. All cases indicate the development of pre-mature stop codons leading to truncated proteins that would be expected to knock out the function of OsPDS. M: methionine amino acid, *: stop/termination codon.

Supplementary Table S1. Summary of the *OsPDS* samples analyzed through target amplicon analysis.

	Template for target amplicon analysis			Target amplicon analysis - sgRNA1				Target amplicon analysis - sgRNA2			
	Total samples analyzed	Genomic DNA	PCR amplicon	Insertion (R1)	Deletion (R1)	Insertion (R2)	Deletion (R2)	Insertion (R1)	Deletion (R1)	Insertion (R2)	Deletion (R2)
**Seeds**	59	37	22	0	3	0	0	0	4	0	12
**SAMs**	18	18	0	0	1	0	0	0	0	0	0
**Pooled seeds**	28	28	0	0	2	0	0	1	0	1	8
**Total**	**105**	**83**	**22**	**0**	**6**	**0**	**0**	**1**	**4**	**1**	**20**

Note: Samples for target sequence analysis were prepared using as template genomic DNA or previously isolated PCR product. Pair-end target amplicon analysis (1 million read pairs (Read Length-250x250)) was performed with a MiSeq and the resulted fastq files were analyzed by CRIS.py. Detection of indels for the respective strands (R1 or R2) of the target sgRNA regions is depicted.

Supplementary Table S2. Primers used for PCR amplification.

Target	Lab Code	Sequence (5′ to 3′)
GFP transcripts (F)	NT068	TGAGGGATACGTGCAGGAG
GFP transcripts (R)	NT069	TGCCGTTCTTTTGCTTGTCG
YFP transcripts (R)	NT076	AAGAAGATGGTGCGCTCCTG
YFP transcripts (F)	NT077	ACGTAAACGGCCACAAGTTC
Elongation Factor 1-α (F)	NT122	TCATCATGAACCACCCTGGC
Elongation Factor 1-α (R)	NT123	TGGGCTTGGTGGGAATCATC
GUSPlus plasmid (F)	NT035	TCCGACCTGATGCAGCTCTC
GUSPlus plasmid (R)	NT036	GATTCCTTGCGGTCCGAATG
GUSPlus transcripts (F)	NT039	GCACCATCAAGACGTTCTCC
GUSPlus transcripts (R)	NT040	CTTCTGTGGGTCGAGTTCCT
OsPDS genomic sequence (F)	NT005	GCTTCGCAAGTAGCAGCATC
OsPDS genomic sequence (R)	NT008	GGTGCAGGCAATGTTTCAGG
GUSPlus cassette amplification (F)	NT057	AAGCTTCACCTGCTCATCCGGTGAGACTTTTCAACAAAGGG
GUSPlus cassette amplification (R)	NT058	GGATCCCACCTGCCCACCACTGATCTAGTAACATAGATGACAC
tRNA-sgRNA targeting *OsPDS* (F)	NT062	AAGCTTCGTCTCTTGGCAACAAAGCACCAGTGGTCTAG
tRNA-sgRNA targeting *OsPDS* (R)	NT063	GGATCCCGTCTCTAAACGCATCTGCCAG
